# Prediction of soil available water-holding capacity from visible near-infrared reflectance spectra

**DOI:** 10.1038/s41598-019-49226-6

**Published:** 2019-09-06

**Authors:** Michael Blaschek, Pierre Roudier, Matteo Poggio, Carolyn B. Hedley

**Affiliations:** 1 Manaaki Whenua – Landcare Research, Private Bag 11052, Manawatu Mail Centre, Palmerston North, 4442 New Zealand; 2 Te Pūnaha Matatini, A New Zealand Centre of Research Excellence, Private Bag 92019, Auckland, 1142 New Zealand

**Keywords:** Environmental sciences, Solid Earth sciences

## Abstract

Sustainable land management requires reliable information about soil hydraulic properties. Among these properties, available water-holding capacity (AWC) is a key attribute, as it quantifies the amount of water available for plants that the soil can hold. Since direct measurements of AWC are costly, pedotransfer functions (PTF) are often used to estimate AWC, leveraging statistical relationships with properties that are easier to measure, such as texture, bulk density, and organic carbon content. This study evaluates visible near-infrared spectroscopy (vis-NIR) as an alternative approach to predict volumetric water content at field capacity (FC) and permanent wilting point (PWP) — AWC being the difference between PWP and FC. A suite of 970 vis-NIR soil spectra, recorded from air-dried, 2-mm, sieved soil samples, were associated with FC and PWP analytical data obtained from New Zealand’s National Soils Database. Partial least squares (PLS) regression and support vector machines on PLS latent variables (PLS-SVM) were used for spectroscopic modelling. With root mean squared errors below 7% and 5% for FC and PWP, respectively, our results indicate that vis-NIR spectroscopy can be used to quantitatively predict volumetric water content at FC and PWP.

## Introduction

Soil data of adequate quality and reasonable quantity is critically important for sustainable land management. This is particularly true in light of an ever increasing pressure on agricultural land, along with rising demands for food production due to global population growth. Agriculture is already by far the biggest user of globally allocated freshwater with over 70% used for irrigation^1^. To improve irrigation efficiency, governing factors that control the storage and release of water in the soil need to be known. Among these factors, available water-holding capacity (AWC) is a key attribute, as it quantifies the amount of water available for plants that the soil can hold. The AWC is defined as the amount of water held by the soil between field capacity (FC) and permanent wilting point (PWP). It is conventionally estimated under controlled laboratory conditions applying a known pressure to a soil core, typically 10–33 kPa (varying between countries) for FC and 1500 kPa for PWP. The method requires dedicated apparatus, and is time and cost consuming. For these reasons, indirect estimation using pedotransfer functions (PTF) is often preferred. PTF leverage the relationships between AWC and soil attributes that are relatively easier to obtain, such as soil texture, bulk density, and soil organic carbon^2–4^. Recently, McNeill *et al*.^5^ presented a set of PTF to predict soil water content at different pressures for New Zealand soils, based predominantly on qualitative predictors such as soil classification and functional horizon designations, which are available from the national soil information system.

Alternatively, it has been suggested that diffuse reflectance spectroscopy could be used to predict soil hydraulic properties directly. This has as advantage that a single, rapid (spectral) reading obtained from a sieved, air-dry sample is used to infer attributes like FC and PWP rather than a set of multiple, lengthy lab measurements used by traditional PTFs. Soil spectroscopy relies on the fact that the fraction of reflected light received by the instrument at any given wavelength (typically between 350 and 2500 nm in the visible and near–infrared parts of the spectrum) is related to vibrational and rotational states of molecules containing oxygen, hydrogen, carbon, or nitrogen atoms. The recorded spectrum is then used to derive a statistical model which relates a soil property to the spectral information. Such spectroscopic models have been derived for a range of soil attributes^6,7^, especially those related to organic molecules or water retention, such as soil organic carbon^8–10^, cation exchange capacity (CEC)^11^ and clay content^12,13^, as well as soil bulk density^14,15^ and saturated soil hydraulic conductivity^16,17^.

However, substantially fewer publications report prediction models for AWC components, i.e. FC and PWP^18,19^. Arslan *et al*.^19^ used visible and near-infrared (vis-NIR) spectroscopy to predict FC and PWP as well as particle size (clay, sand and silt content) for 305 soil samples collected within a study area of 8,000 ha in the Bafra plain, Turkey. They compared a number of different regression models, and concluded that multiple regression models provided best prediction results. The spectral reflectance at 350–420 nm was correlated positively with both FC and PWP, whereas spectral reflectance in the 500–2,500 nm region was negatively correlated. Excellent predictions were obtained for PWP, while predictions for sand were poor, reflecting the importance of large surface area and moisture for relating spectral reflectance to soil attributes. A similar study by Viscarra Rossel and Webster^18^ used vis-NIR spectra recorded from 20,000 soil samples of soil collected across Australia to predict a wide range of properties, including FC and PWP. The authors used a decision tree approach, Cubist, to develop prediction models. They report prediction of FC and PWP, but do not provide specific details about contributing wavelengths.

Very recently, Pittaki-Chrysodonta *et al*.^20^ investigated the ability of vis-NIR spectra for predicting the Campbell soil water retention function and compared their results with classical PTF. The authors conclude that predicting the pore-size distribution parameter of the Campbell function as well as volumetric water content at 100 kPa by vis-NIR spectra provided very good approximations of measured soil water retention data for various agricultural fields in Denmark. However, their study was limited to soils with percentages of clay fraction plus organic matter content below 20%, which is what they define as soil fines.

Mid-infrared (MIR) diffuse reflectance spectroscopy has also been used to predict soil hydraulic properties^21,22^. It has the advantage that it tends to provide slightly improved prediction accuracy for soil attributes (e.g. clay, carbon and CEC) than vis-NIR, but the disadvantage that it is more labour intensive (requiring fine grinding of the sample), and records data from a very small sample size. Minasny *et al*.^21^ and Tranter *et al*.^22^ found MIR spectroscopy to be a valuable predictor of fundamental soil constituents, but less so for moisture retention values. They recommend, instead, that MIR be used in conjunction with PTF to improve moisture retention predictions.

In this study, our goal is to ascertain how well the New Zealand vis-NIR spectral library, recorded from 2-mm sieved, air-dry soils stored in the New Zealand Soils Archive, can be used to directly predict water contents at FC and PWP for soils across the country. In doing so, we propose an approach that indirectly exploits the soil water relationships with clay content, clay mineralogy and soil organic carbon content by statistically analysing the differences in shape and intensity of diffuse reflectance spectra measured from sieved, air-dry soils. Successful direct spectral prediction models for FC and PWP would enable higher density sampling for improved modelling, for example to produce AWC maps to better inform soil management practices such as irrigation planning and for agro-environmental assessments of climate change impacts on food production, because soil type-related yield variability generally outweighs simulated variability due to weather^23^. National calibration models of PWP and FC and, by difference estimates of AWC, provide an innovative approach to address these national environmental modelling imperatives.

## Results and Discussion

### Performance of the FC and PWP predictions

**Figure 1:**
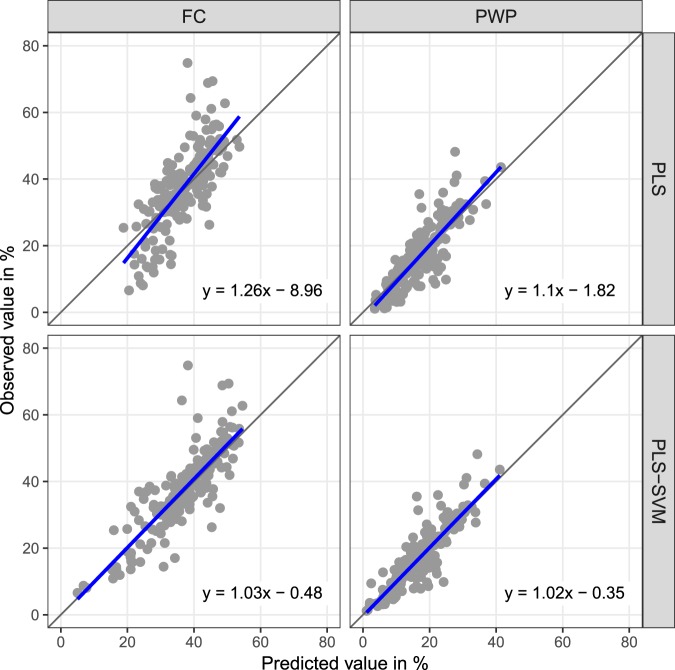
Scatterplots of actual versus predicted values for volumetric water content at field capacity (FC) and permanent wilting point (PWP) using PLS regression and PLS-SVM models. Solid lines refer to linear regression models of observations on estimates for validation data (N = 186). Dashed lines are (ideal) 1:1 lines.

Partial least squares (PLS) models were calibrated for FC and PWP showing optimal numbers of latent variables of 16 and 18, respectively. Figure 1 illustrates the performances of the PLS regressions and PLS-SVM models calculated on the validation set. Results show little bias (less than 0.7%). The PLS-SVM prediction model for PWP outperformed that for FC (RMSE = 4.41 and 6.68%, *R*^2^ = 0.78 and 0.7, RPD = 2.12 and 1.81, respectively, on the validation set). Although the distribution of measured versus predicted FC and PWP values shows that most validation points are evenly distributed around the 1:1 line, the FC model exhibits some under-predictions of high values. Using SVM on the latent variables, as opposed to a linear model, mitigated the impact of non-linear relationships, and significantly helped reduce prediction errors. Table 1 provides summary statistics for FC and PWP calibrations, calculated from validation data using PLS-SVM. It also shows summary for AWC estimates obtained by subtracting the PWP from the FC predictions.

**Table 1:** Overview statistics and performance measures for volumetric water content (%) at field capacity (FC) and permanent wilting point (PWP) calculated from the validation set using PLS-SVM as well as resulting available water-holding capacity (AWC) estimates.

Attr.	N	Min (%)	Max (%)	Mean (%)	SD (%)	RMSE (%)	*R* ^2^	RPD	Bias (%)
FC	186	5.02	54.6	36.8	9.82	6.68	0.70	1.81	−0.67
PWP	186	1.08	41.1	17.0	7.94	4.41	0.78	2.12	−0.06
AWC	186	5.24	34.8	19.9	5.93	4.86	0.58	1.54	−0.61

### Vis-NIR spectra for FC and PWP modelling

**Figure 2:**
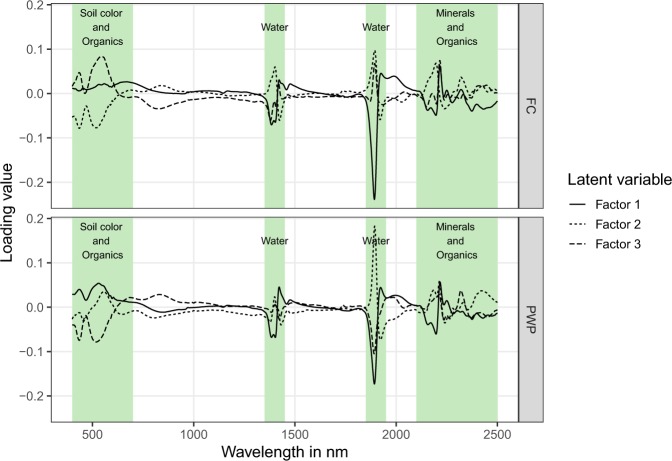
PLS regression loadings for the first three latent variables for volumetric water content at field capacity (FC) and permanent wilting point (PWP). Coloured background represents wavelength regions of particular importance for the models.

Figure 2 shows the PLS loadings for the first three latent variables of each PLS prediction model. For the PWP prediction model, the first factor accounts for 52.5% of the spectral variation. Another 25% is added by the latent variables 2 (9.9%) and 3 (15.5%). For FC the total amount of spectral variation explained by the first three factors is similar (78.3%). However, unlike for PWP, latent variable 1 explains only 42.2% for FC with latent variables 2 and 3 adding another 25.3% and 10.8%, respectively. These loadings provide insights on the relative contribution of each wavelength to a particular prediction model. For both models, the most influencing wavelengths in the context of the first three latent variables were found around 500 nm, at 1400 nm and beyond 2000 nm. Bands in the visible portion (400–700 nm) of the electromagnetic spectrum are related to soil colour and, therefore, may correspond to iron minerals and to a weaker extent to soil organic matter^24^. Important absorption features at 1400 nm and between 2100 and 2500 nm usually refer to metal-OH bending and OH stretching modes and are often attributed to the amount and type of clay minerals^7,25,26^. By detecting the mineral and organic components of the solid soil particles, soil spectroscopy can indirectly estimate both, water content at FC and PWP, because of their special link with clay and carbon content.

### Performance of the models across soil orders

**Figure 3:**
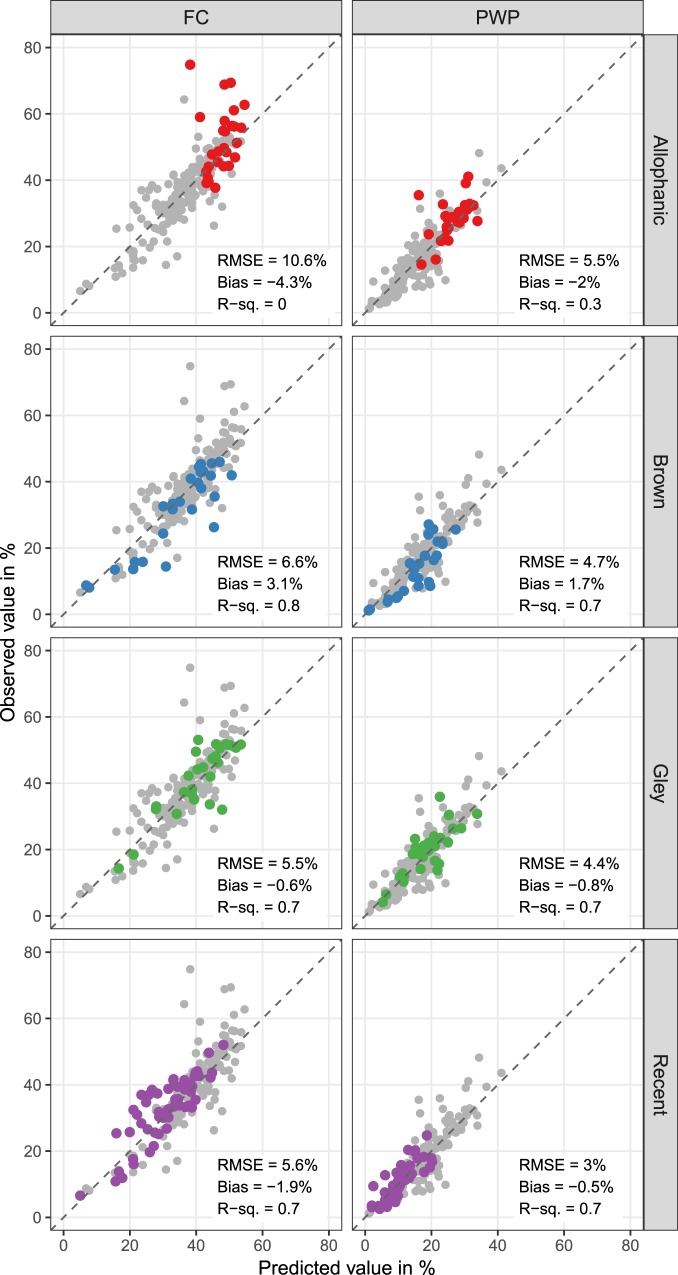
Scatterplots of actual versus PLS-SVM predicted values for volumetric water content at field capacity (FC) and permanent wilting point (PWP) by soil order. Dashed lines are (ideal) 1:1 lines. Only the most common soil orders are shown. Soils are classified in accordance with the New Zealand Soil Classification^27^. Coloured dots are the validation samples that belong to the respective soil orders. Grey dots are the remaining samples of the validation set.

Figure 3 shows the predicted versus the observed values for FC and PWP for 4 of the most abundant soils of the 15 soil orders, the highest level in the New Zealand Soil Classification (NZSC)^27^: Allophanic soils (Andosols in the FAO-WRB classification system^28^), Brown soils (Cambisols/Arenosols), Gley soils (Gleysols/Fluvisols), and Recent soils (Fluvisols/Regosols/Arenosols/Leptosols).

Close examination of this figure shows that the soils with the highest FC values which are poorly predicted by the model are mostly Allophanic soils. One possible explanation for the difficulties encountered by the model to adequately predict FC for Allophanic soils, typically of volcanic origin, is that these soils are more likely to have conventional lab analysis measurement error due to their specific characteristics. Allophanic soils have a high capacity for water retention associated with very high specific surface area. When FC is measured on a soil kept in its field moist state, FC is generally greater than 100% of the weight of dry soil (at 105 °C). It can reach 300%; however, if the soil dehydrates this dehydration is irreversible (hysteresis effect)^29^. This will influence lab estimations of FC depending on whether FC measurements were undertaken on a field moist sample or on the same soil sample after drying and re-wetting. Therefore, the estimated FC values for Allophanic soils are likely to have a larger uncertainty than for other soil orders.

Another problematic soil order are Brown soils (N = 26), whose FC model is over-predicting lower values (positive bias of 3.1% for FC and 1.7% for PWP). Although Brown soils, some of which contain a minor allophanic component, represent a very large, heterogeneous group within the NZSC, it is difficult to suggest a specific reason for their systematic over-estimation. On the other hand, very good results are obtained on Recent soils (N = 53). These soils are of particular interest due to their importance for agricultural use in New Zealand, which justifies a specific focus in our study.

### Comparison with published prediction models

Only a few studies have published attempts to use vis-NIR spectroscopy to predict directly volumetric water content at FC and PWP. One such attempt is described in Arslan *et al*.^19^, who obtained good results using PLS regression on 305 soil spectra for FC (*R*^2^ = 0.77; RMSE = 5.24%; RPD = 1.81) and PWP (*R*^2^ = 0.78; RMSE = 3.87%; RPD = 2.00). Viscarra Rossel and Webster^18^ also reported predictions of FC (RMSE = 0.06 *m*^3^/*m*^3^; RPD = 1.68) and PWP (RMSE = 0.04 *m*^3^/*m*^3^; RPD = 1.95) based on vis-NIR data from the Australian soil spectral library using the machine learning algorithm Cubist. The results of our water content models are very similar to those obtained by these authors in terms of average errors (FC: RMSE = 6.68%, RPD = 1.81; PWP: RMSE = 4.41%, RPD = 2.12).

The models reported in this study outperform the ones obtained by the most recent PTF estimates for New Zealand soils, as reported by Cichota *et al*.^4^ (PWP: RMSE = 5.6%) and on a very similar dataset by McNeill *et al*.^5^ (FC: RMSE = 7.6%, *R*^2^ = 0.6; PWP: RMSE = 4.9%, *R*^2^ = 0.78). This difference can be interpreted by the fact that McNeill *et al*.^5^ used mainly qualitative predictors. In particular, their inference system does not have access to quantitative estimates of critical covariables for FC and PWP, such as carbon or clay, while the correlation of these attributes with vis-NIR spectroscopy is well documented. This difference in performance illustrates the potential of vis-NIR spectroscopy to be included as a routine measurement in national soil information systems.

### Implications and limitations

PLS-SVM models, calibrated for water content at field capacity and permanent wilting point, were successfully tested with RMSE values of 6.68% and 4.41%, respectively. Hence, our results indicate that vis-NIR spectroscopy can be used to model AWC components, and that SVM regression after data compression based on PLS factors is an effective and efficient tool to do so.

While overall performances are promising, and critical New Zealand soil orders (such as Recent soils) are well predicted, there are a few exceptions that need further attention. In particular, soils classified as Allophanic soils were found difficult to predict, particularly when modelling water content at field capacity. This is likely related to the fact that Allophanic soils are characterised by amorphous clay minerals of variable charge that undergo irreversible physical changes during drying which induce micro-aggregation of the soil which would not normally occur. This causes repeatability problems for lab estimations of particle size analysis and field capacity of these soils^30,31^. Future research will aim to enhance the prediction models for PWP and FC by including morphological soil features obtained, for example, from image analysis of the soil surface. Using our national scale models to eventually develop site-specific calibrations in a region known to be dominated by this type of soil will require adjustments based on local samples. This could be implemented using any memory-based learning method^32^, an augmenting approach like spiking^33^ or by more recent techniques based on resampling, such as RS-LOCAL^34^. In addition to issues introduced by varying soil orders, possible limitations associated with model robustness over different spatial extents need to be analysed^26^.

By providing nation-wide prediction models for water content at FC and PWP this work sets the stage for a quick and accurate determination of available water-holding capacity. Moreover, it provides the opportunity to generate large datasets that in turn can be used to produce a new generation of high resolution maps of AWC, given this soil attribute is key to sustainable land management and effective irrigation planning.

## Methods

**Figure 4:**
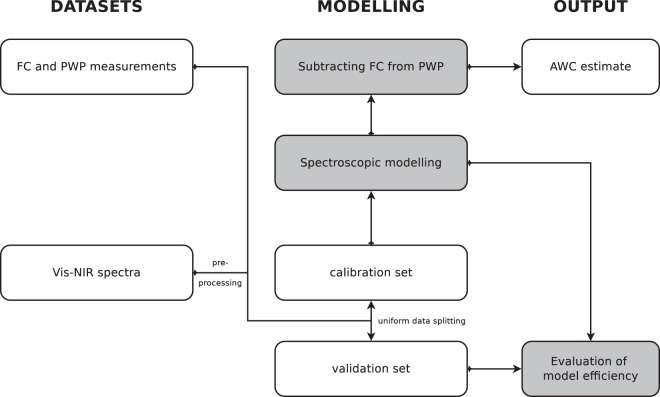
Flow chart showing the AWC modelling from spectra.

Substantial steps of the vis-NIR modelling exercise are summarised in Figure 4.

### Soil properties

The New Zealand Soils Archive is a physical collection of air-dried soil samples sieved finer than 2 mm, located in Palmerston North, New Zealand. The Archive stores samples collected across the country from the mid-1940s. For this study, soil samples that have volumetric water content recorded at two different pressure heads were selected, with their analytical data extracted from New Zealand’s National Soils Database. Water content for pressure potentials at 10 kPa (FC) and 1500 kPa (PWP) were determined by controlled drainage methods using intact soil cores (ring size = 100 mm diameter, 75 mm deep) for FC and repacked 2-mm sieved soil in rings (10 mm height by 50 mm diameter) for PWP^35^. Due to unreliable estimates of soil water content (for explanation see, e.g. Huntington^36^), samples were eliminated if soil bulk density observations were below 0.5 *Mg*/*m*^3^. Samples from three soil profiles classified as Organic soils (Histosols in the FAO-WRB classification system^28^) were excluded. The total number of samples available for analysis was 970, from 210 unique profiles. The maximum soil depth found was 175 cm. 147 samples were taken from the soil surface layer.

**Figure 5:**
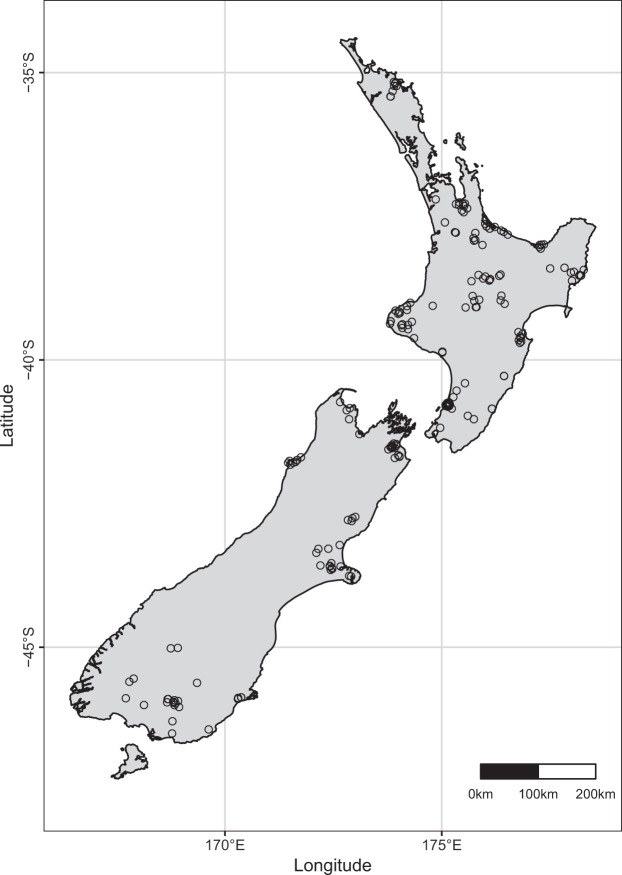
Location of the soil samples used to build the spectral library. The sampling scheme reflects the location of the major agricultural regions in New Zealand.

Figure 5 shows the profile location of the retained samples. All major agricultural areas of New Zealand were captured by the sampled soils. Soil preparation and laboratory analyses were undertaken at the Manaaki Whenua – Landcare Research Environmental Chemistry Laboratory (located in Palmerston North, New Zealand), or its predecessor, the New Zealand Soil Bureau Laboratory (Soil Bureau, DSIR, Department of Scientific and Industrial Research, Lower Hutt, New Zealand).

**Figure 6:**
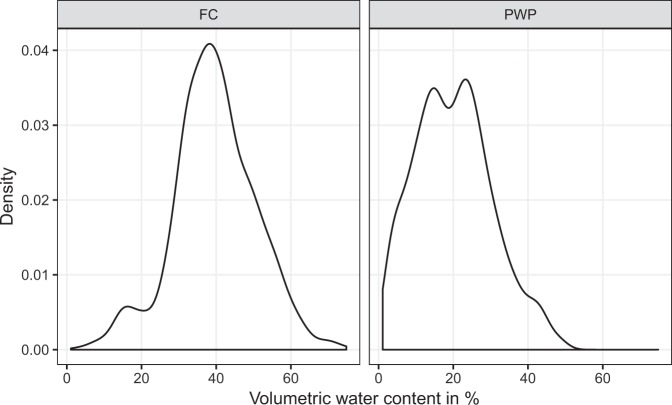
Probability density functions of measured volumetric water content at field capacity (FC) and permanent wilting point (PWP) (N = 970).

The sample set has a comprehensive range of measured volumetric water content at field capacity (FC) from 2.75% to 75%, with a mean value of 39% and a standard deviation of 11%. Volumetric water content at permanent wilting point (PWP) varied from 1.10% to almost 50%, with a mean value of 20% and a standard deviation of 10% (see Table 2). The large variations observed for both FC and PWP reflect the diversity of soils represented in the National Soils Database. Figure 6 shows the probability density functions for FC and PWP. While FC is rather symmetric (skewness ≈ 0), the distribution of PWP is slightly right-skewed (positive skewness, but still inferior to 1). Common transformations, such as square root and log-transformation, did not improve skewness for PWP.

**Table 2:** Summary statistics of observed volumetric water content (%) at field capacity (FC) and permanent wilting point (PWP) as well as clay, silt and sand content, and soil organic carbon (SOC) content.

Attr.	N	Min.	Max.	Mean	SD	IQR	Skew.	Kurt.
FC	970	2.75	74.85	39.55	11.04	13.70	−0.08	0.44
PWP	970	1.10	49.25	20.12	10.26	14.35	0.36	−0.36
Clay	350	0.00	66.00	24.34	11.91	16.00	0.18	−1.90
Silt	350	2.93	90.79	50.12	12.99	17.12	−0.19	−1.60
Sand	350	0.00	97.07	25.53	19.22	27.21	0.34	−1.64
SOC	948	0.07	20.60	2.28	2.61	2.62	2.07	5.61

### Spectral library

**Figure 7:**
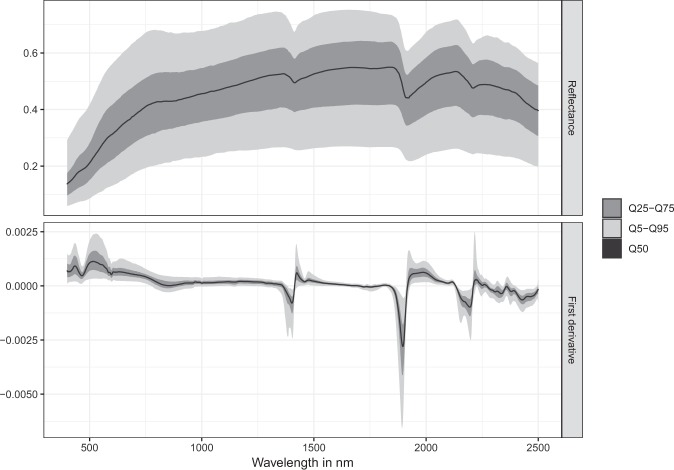
Distribution of spectral reflectance values and first order derivatives showing intervals defined by the 5th, 25th, 75th and 95th percentiles (N = 970). Q50 denotes the median soil spectra.

The New Zealand soil spectral library was developed from soils stored in the National Soils Archive, using an ASD FieldSpec 3 spectroradiometer (Analytical Spectral Devices Inc., Boulder, Colorado, USA) with an ASD High Intensity Contact Probe, in bench mode. The contact probe has a 10 mm spot size and uses a high intensity 4.5 W Halogen bulb as light source. Air-dried and 2 mm sieved soil samples were presented to the contact probe in a Petri dish (10 mm height by 85 mm diameter). The device records soil reflectance at wavelengths ranging from 350 to 2500 nm at a nominal spectral resolution of 3 nm at 700 nm and 10 nm at 1400/2100 nm, that is then interpolated to 1 nm by the device’s software. A white reference measurement was made every 10 spectra using a piece of Spectralon. The reflectance spectra recorded for each sample was an average of 50 internal readings and sample positions were held constant for the duration of the experiments. Figure 7 summarises the average spectra by showing the 5th, 25th, 50th, 75th and 95th percentiles of spectral reflectance before and after pre-processing.

### Spectral pre-processing

Reflectance spectra were exported from the FieldSpec unit, and imported into R^37^ for further analysis. The spectra were spliced in an additive manner^38^ [p.17], to remove the steps introduced at the junction between the 3 detectors (VNIR, SWIR1, and SWIR2) used to cover the vis-NIR spectral range. The noisiest part of the spectral range was excluded (350–400 nm). The Savitzky-Golay filter^39^ was used to derive and smooth the spectra (first derivative, with a filter length and order of 11 and 2, respectively).

Spectral information was further reduced for data exploration, outlier detection, and data splitting using principal component analysis (PCA). The singular value decomposition method was used, and spectra were centered before factorization. The first three principal components were retained, and accounted for 80% of the overall variance. Based on a quantile threshold of 97.5% on the Mahalanobis distances calculated from the principal components, 39 spectra were considered as outliers, and discarded from further analysis.

### Spectroscopic modelling

The Kennard–Stone algorithm^40^ was used to uniformly split the remaining 931 spectra into 745 samples used for model calibration, and a validation set of 186 samples not used for model development. Partial least squares (PLS)^41^ regression was used for spectroscopic modelling, as implemented in the pls package^42^. The caret package^43^ was used to identify the optimal number of latent variables, based on the one standard error rule from Breiman *et al*.^44^. To avoid over-fitting, this heuristic approach implements a parsimony rule, and selects the simplest model within one standard error of the optimal model determined from the cross-validated root mean square error (RMSE) on the calibration dataset. The calibration step used repeated 10-fold cross-validation with 30 repeats and randomly generated segments.

To account for possible non-linear effects between AWC parameters and the spectra, an additional set of models were calibrated based on the first 16 and 18 latent variables of the PLS models derived for FC and PWP, respectively. These retained latent variables were centered and scaled, then used as predictors for a support vector machines (SVM) regression. SVMs for real-valued function estimation problems were introduced by Vapnik^45^ offering a new non-linear regression technique based on the SVM framework which was rapidly adopted in the field of vis-NIR spectroscopy^46^. If applied to PLS factors rather than spectral wavelengths, SVM regression models were found to be more robust^47^, since PLS removes some of the unwanted noise inherent in reflectance spectra. The SVM models used in this study leverage Gaussian radial basis function (RBF) kernels, as implemented in the kernlab package^48^. For a constant epsilon (margin of tolerance) of 0.1, the remaining hyper-parameters sigma (inverse kernel width) and C (regularization term to fight over-fitting) were tuned using caret^43^, based on custom 10 times 10 grid searches running repeated 10-fold cross-validation with 30 repeats. The SVM model selection of hyper-parameters was made based on lowest RMSE values.

### Model evaluation

Model performance was quantified by metrics calculated on samples from the validation set. These samples were not used for calibration of the models. Those metrics were the root mean squared error (RMSE), the coefficient of determination from regression between estimated and observed values (*R*^2^), the ratio of performance to deviation (RPD, i.e. the ratio of the standard deviation of the sample to the standard error of prediction), and the bias.

## Data Availability

The datasets analysed during the current study are available from the corresponding author on reasonable request.

## References

[1] Fischer G, Tubiello FN, van Velthuizen H, Wiberg DA (2007). Climate change impacts on irrigation water requirements: Effects of mitigation, 1990–2080. Technological Forecasting and Social Change.

[2] Hall, D. G. M., Reeve, M. J., Thomasson, A. J. & Wright, V. F. Water retention, porosity and density of field soils. Tech. Rep. N9, Soil Survey of England and Wales (1977).

[3] Minasny B, McBratney AB, Bristow KL (1999). Comparison of different approaches to the development of pedotransfer functions for water-retention curves. Geoderma.

[4] Cichota R, Vogeler I, Snow VO, Webb TH (2013). Ensemble pedotransfer functions to derive hydraulic properties for New Zealand soils. Soil Research.

[5] McNeill SJ, Lilburne LR, Carrick S, Webb TH, Cuthill T (2018). Pedotransfer functions for the soil water characteristics of New Zealand soils using S-map information. Geoderma.

[6] McBratney AB, Minasny B, Viscarra Rossel R (2006). Spectral soil analysis and inference systems: a powerful combination for solving the soil data crisis. Geoderma.

[7] Stenberg Bo, Viscarra Rossel Raphael A., Mouazen Abdul Mounem, Wetterlind Johanna (2010). Visible and Near Infrared Spectroscopy in Soil Science. Advances in Agronomy.

[8] Kusumo BH (2008). The use of diffuse reflectance spectroscopy for *in situ* carbon and nitrogen analysis of pastoral soils. Soil Research.

[9] Minasny Budiman, McBratney Alex B., Malone Brendan P., Wheeler Ichsani (2013). Digital Mapping of Soil Carbon. Advances in Agronomy.

[10] Viscarra Rossel RA, Brus DJ, Lobsey C, Shi Z, McLachlan G (2016). Baseline estimates of soil organic carbon by proximal sensing: comparing design-based, model-assisted and model-based inference. Geoderma.

[11] Cañasveras Sánchez, J. C., Barrón, V., del Campillo, M. G. & Viscarra Rossel, R. A. Reflectance spectroscopy: a tool for predicting soil properties related to the incidence of Fe chlorosis. *Spanish journal of agricultural research* 1133–1142 (2012).

[12] Sørensen LK, Dalsgaard S (2005). Determination of clay and other soil properties by near infrared spectroscopy. Soil Science Society of America Journal.

[13] Lagacherie P, Baret F, Féret J-B, Netto JM, Robbez-Masson JM (2008). Estimation of soil clay and calcium carbonate using laboratory, field and airborne hyperspectral measurements. Remote Sensing of Environment.

[14] Moreira CS (2009). Near infrared spectroscopy for soil bulk density assessment. European Journal of Soil Science.

[15] Roudier P, Hedley CB, Ross CW (2015). Prediction of volumetric soil organic carbon from field-moist intact soil cores. European Journal of Soil Science.

[16] Santra P (2009). Estimation of soil hydraulic properties using proximal spectral reflectance in visible, near-infrared, and shortwave-infrared (VIS–NIR–SWIR) region. Geoderma.

[17] Xu C (2017). Enhancing pedotransfer functions (PTFs) using soil spectral reflectance data for estimating saturated hydraulic conductivity in southwestern china. Catena.

[18] Viscarra Rossel RA, Webster R (2012). Predicting soil properties from the australian soil visible–near infrared spectroscopic database. European Journal of Soil Science.

[19] Arslan H, Tasan M, Yildirim D, Koksal ES, Cemek B (2014). Predicting field capacity, wilting point, and the other physical properties of soils using hyperspectral reflectance spectroscopy: two different statistical approaches. Environmental monitoring and assessment.

[20] Pittaki-Chrysodonta Zampela, Moldrup Per, Knadel Maria, Iversen Bo V., Hermansen Cecilie, Greve Mogens H., de Jonge Lis Wollesen (2018). Predicting the Campbell Soil Water Retention Function: Comparing Visible–Near-Infrared Spectroscopy with Classical Pedotransfer Function. Vadose Zone Journal.

[21] Minasny B, McBratney AB, Tranter G, Murphy BW (2008). Using soil knowledge for the evaluation of mid-infrared diffuse reflectance spectroscopy for predicting soil physical and mechanical properties. European Journal of Soil Science.

[22] Tranter G, Minasny B, McBratney AB, Viscarra Rossel RA, Murphy BW (2008). Comparing spectral soil inference systems and mid-infrared spectroscopic predictions of soil moisture retention. Soil Science Society of America Journal.

[23] Folberth C (2016). Uncertainty in soil data can outweigh climate impact signals in global crop yield simulations. Nature communications.

[24] Viscarra Rossel RA, Chappell A, De Caritat P, McKenzie NJ (2011). On the soil information content of visible–near infrared reflectance spectra. European Journal of Soil Science.

[25] Ben-Dor E, Irons JR, Epema GF (1999). Soil reflectance. Remote Sensing of the Earth Sciences: Manual of Remote Sensing.

[26] Gomez C, Coulouma G (2018). Importance of the spatial extent for using soil properties estimated by laboratory VNIR/SWIR spectroscopy: Examples of the clay and calcium carbonate content. Geoderma.

[27] Hewitt, A. E. New Zealand soil classification. 3rd ed. Lincoln, New Zealand. *Manaaki Whenua Press* (2010).

[28] IUSS Working Group WRB. World reference base for soil resources 2014, update 2015: International soil classification system for naming soils and creating legends for soil maps. *World Soil Resources Reports No*. *106*. *FAO*, *Rome* (2015).

[29] Leamy, M. L., Smith, G. D., Colmet-Daage, F. & Otowa, M. Chapter 2 – The morphological characteristics of Andisols. In Theng, B. K. G. (ed.) *Soils with variable charge*, 17–34 (1980).

[30] Maeda T, Warkentin BP (1975). Void changes in allophane soils determining water retention and transmission. Soil Science Society of America Journal.

[31] Singleton PL, Addison B, Boyes M (1999). Differences in particle density between field-moist and oven-dry samples from allophanic soils. Soil Research.

[32] Ramirez-Lopez L (2013). The spectrum-based learner: A new local approach for modeling soil vis–nir spectra of complex datasets. Geoderma.

[33] Guerrero C, Zornoza R, Gómez I, Mataix-Beneyto J (2010). Spiking of NIR regional models using samples from target sites: Effect of model size on prediction accuracy. Geoderma.

[34] Lobsey CR, Viscarra Rossel RA, Roudier P, Hedley CB (2017). RS-LOCAL data-mines information from spectral libraries to improve local calibrations. European Journal of Soil Science.

[35] Gradwell, M. W. & Birrell, K. S. Part C. Methods for Physical Analysis of Soils. *New Zealand Soil Bureau Scientific Report 10C* (1979).

[36] Huntington, T. G. *Available Water Capacity and Soil Organic Matter*, vol. 1, 139–143, 2 edn. (2006).

[37] R Core Team. *R: A Language and Environment for Statistical Computing*. R Foundation for Statistical Computing, Vienna, Austria (2017).

[38] Danner, M., Locherer, M., Hank, T. & Richter, K. Spectral Sampling with the ASD FieldSpec 4 – Theory, Measurement, Problems, Interpretation, 10.2312/enmap.2015.008 (2015).

[39] Savitzky A, Golay MJE (1964). Smoothing and differentiation of data by simplified least squares procedures. Analytical chemistry.

[40] Kennard RW, Stone LA (1969). Computer aided design of experiments. Technometrics.

[41] Wold S, Sjöström M, Eriksson L (2001). PLS-regression: a basic tool of chemometrics. Chemometrics and intelligent laboratory systems.

[42] Mevik, B.-H., Wehrens, R. & Liland, K. H. pls: Partial Least Squares and Principal Component Regression. R package version 2.6-0, https://CRAN.R-project.org/package=pls (2016).

[43] Kuhn, M. Caret: Classification and Regression Training. R package version 6.0-78, https://CRAN.R-project.org/package=caret (2017).

[44] Breiman, L., Friedman, J., Stone, C. J. & Olshen, R. A. *Classification and regression trees* (CRC press, 1984).

[45] Vapnik, V. Chapter 3 – The Support Vector Method of Function Estimation. In Suykens, J. A. K. & Vandewalle, J. (eds) *Nonlinear Modeling: Advanced Black-Box Techniques*, 55–86 (1998).

[46] Chauchard F, Cogdill R, Roussel S, Roger JM, Bellon-Maurel V (2004). Application of LS-SVM to non-linear phenomena in NIR spectroscopy: development of a robust and portable sensor for acidity prediction in grapes. Chemometrics and Intelligent Laboratory Systems.

[47] Bao N, Wu L, Ye B, Yang K, Zhou W (2017). Assessing soil organic matter of reclaimed soil from a large surface coal mine using a field spectroradiometer in laboratory. Geoderma.

[48] Karatzoglou A, Smola A, Hornik K, Zeileis A (2004). kernlab – An S4 Package for Kernel Methods in R. Journal of statistical software.

